# Supplementary material for: Shared genetic aetiology between cognitive performance and brain activations in language and math tasks
Source: Sci Rep. 2018 Dec 4;8:17624. doi: 10.1038/s41598-018-35665-0 (PMC6279777; doi:10.1038/s41598-018-35665-0)
Supplement: Supplementary file 1 — Supplementary Materials [file 41598_2018_35665_MOESM1_ESM.pdf]

Shared genetic aetiology between cognitive performance and brain activations in language and math tasks

Authors:

Yann Le Guen[1], Marie Amalric[2], Philippe Pinel[2], Christophe Pallier[2], Vincent Frouin[1*]*

Affiliations:

[1]Neurospin, Institut Joliot, CEA, Université Paris-Saclay, Gif-sur-Yvette, France
[2]Cognitive Neuroimaging Unit, U992, INSERM, Neurospin, Institut Joliot, CEA, Université Paris-Saclay, Gif-sur-Yvette, France

*** Corresponding authors' information:**

Emails: yann.leguen@cea.fr (YLG), vincent.frouin@cea.fr (VF)

Supporting Information

Supplementary Figures followed by Supplementary Tables.

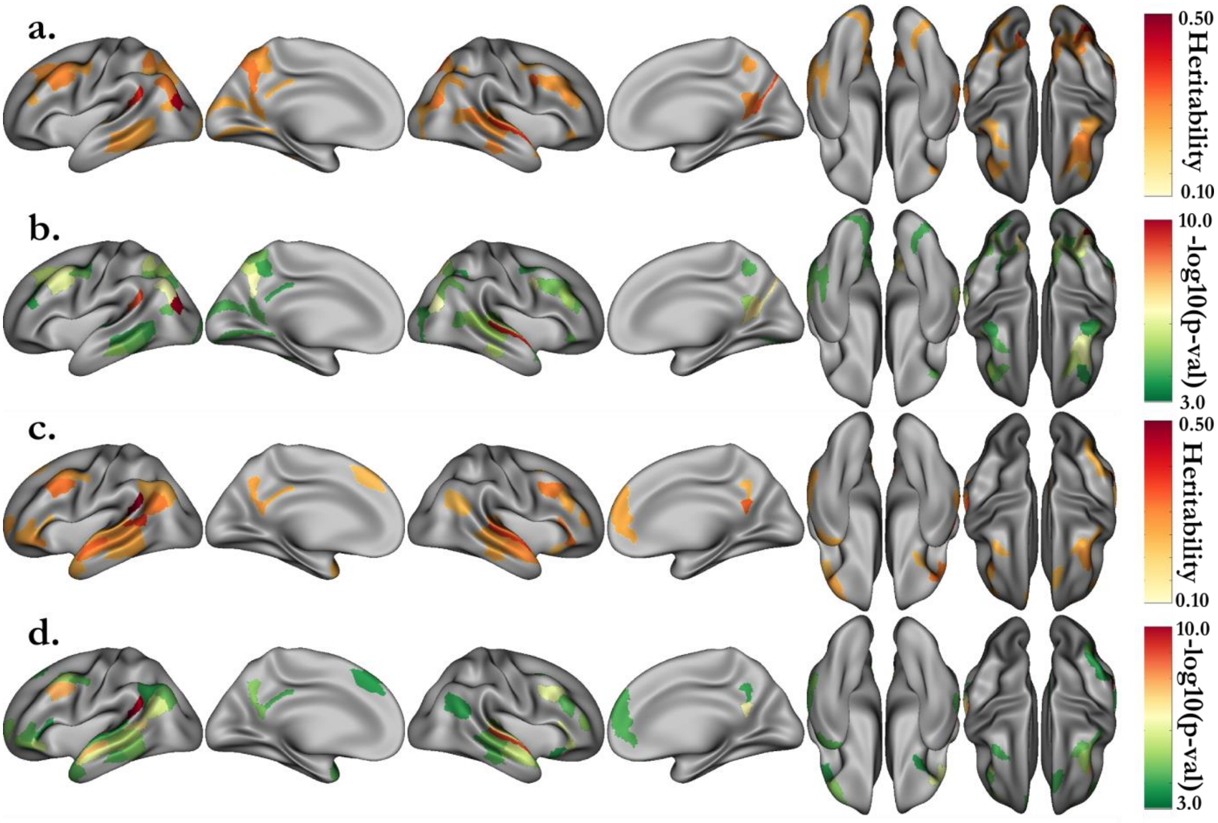

S1 Fig. Heritability estimates for the activations of the tasks MATH (a.) and STORY (c.), and their associated p-values (respectively b., d.), using median z-stat per areal. Only the estimates significant after correction ($p < 0.05/360$, with 180 areals in each hemisphere) are displayed. Activations correspond to the median z-stat in each areal of the HCP multimodal parcellation. There is almost no difference with Fig. 2, when using the median parameter estimate (β) as a proxy for the activation in each areal.

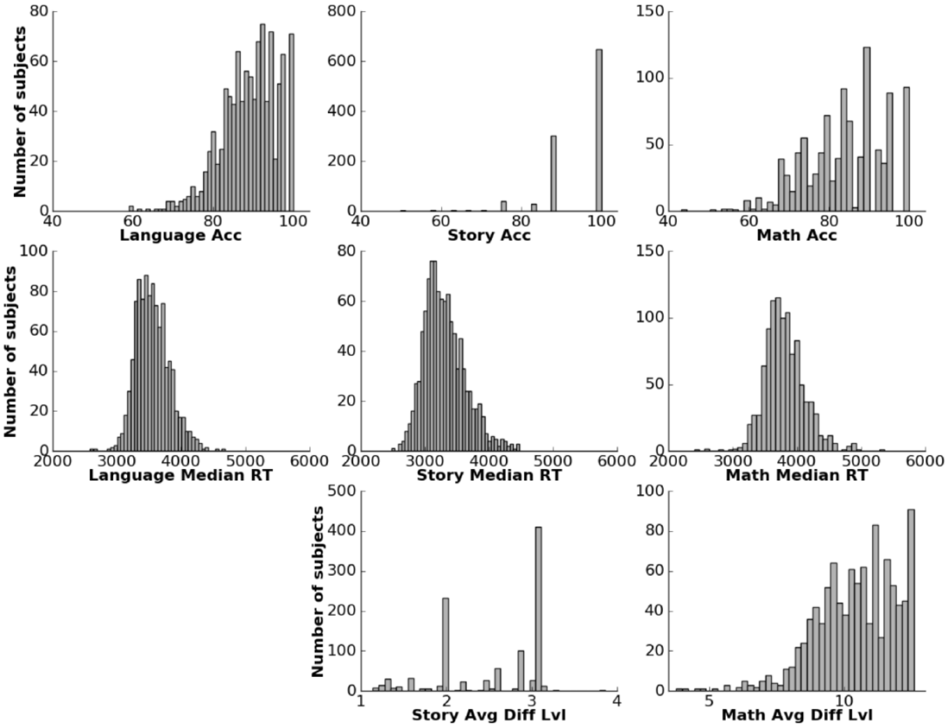

S2 Fig. Distribution of behavioral scores collected by HCP during the language fMRI task, composed of MATH and STORY tasks.

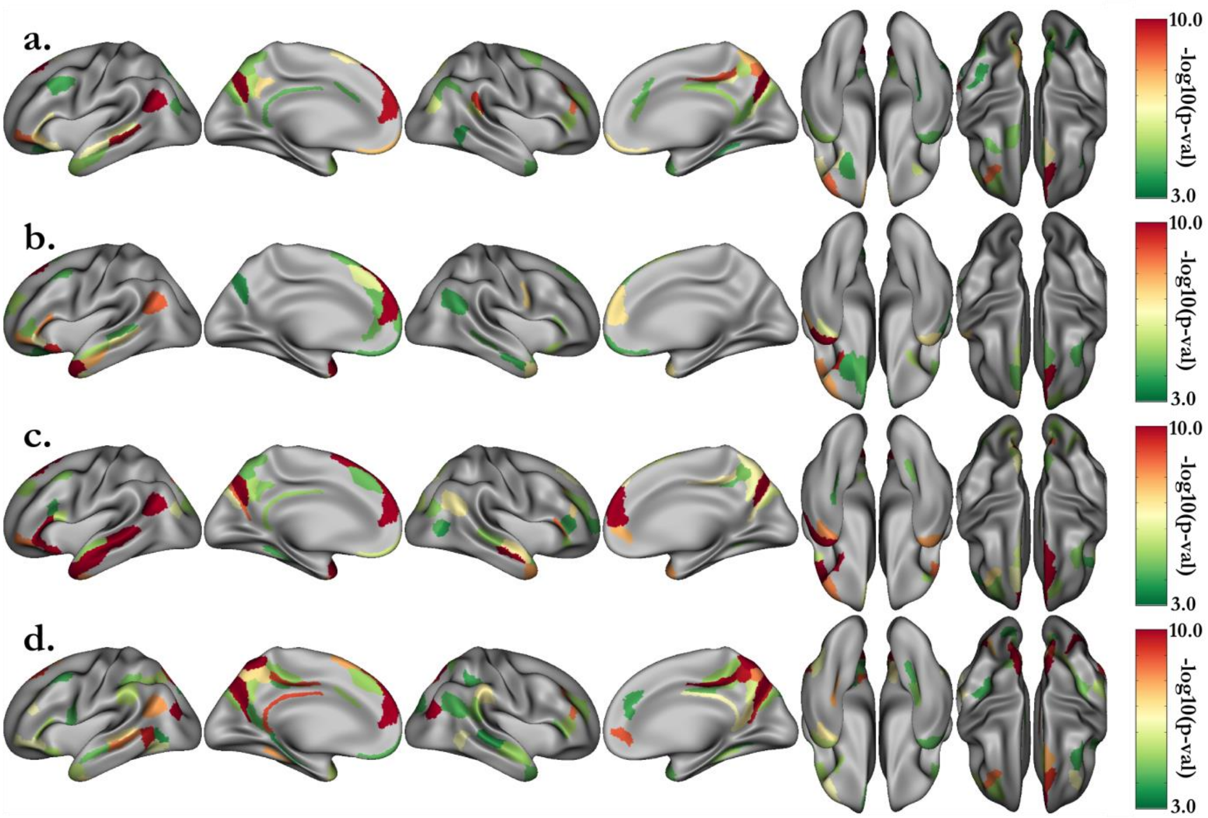

S3 Fig. P-values associated to the phenotypic correlation (Figure 4) between the grayordinates activations of the STORY-MATH contrast and the NIH behavioral scores. a. Fluid Intelligence (PMAT24_A_CR). **b.** Working Memory (ListSort). **c.** Vocabulary Comprehension (PicVocab). **d.** Reading Decoding (ReadEng). All p-values < 0.05/360, Bonferroni correction**.**

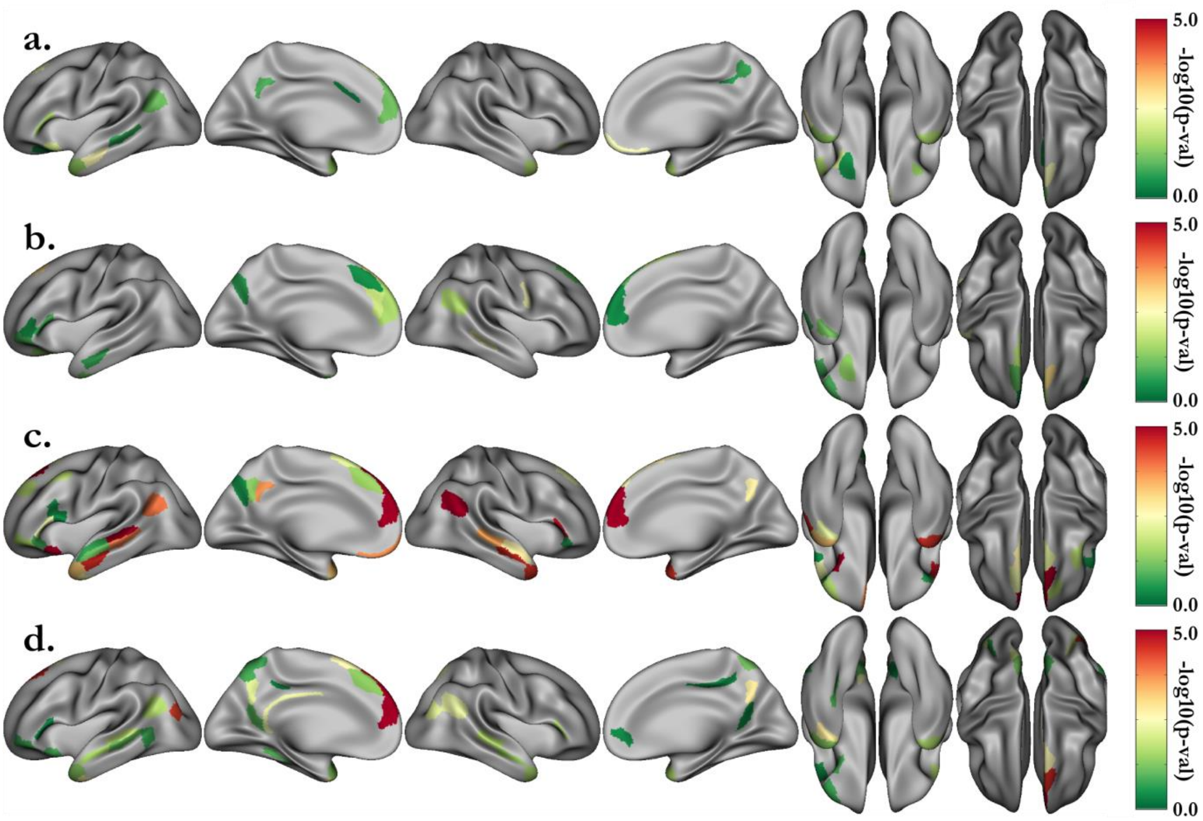

S4 Fig. P-values associated to the genetic correlation (Figure 5) between the grayordinates activations of the STORY-MATH contrast and the NIH behavioral scores. a. Fluid Intelligence (PMAT24_A_CR). **b.** Working Memory (ListSort). **c.** Vocabulary Comprehension (PicVocab). **d.** Reading Decoding (ReadEng). All p-values $p < 0.05$, uncorrected.

S1 Table. Heritability estimates for the NIH behavioral variables. see Methods for description of the analysis.

Trait	h^2±SE (p)	Age	Age^2	Sex	Age*Sex	Age^2*Sex	Hispanic	Educ	h^2cov%
		p-val							
**Fluid Intelligence CR**	0.43±0.06 ($5.8·10^{-13}$)	0.2	0.48	$3.6·10^{-5}$	0.21	0.24	0.55	$1.0·10^{-10}$	9.1
**Fluid Intelligence RT**	0.22±0.06 ($1.0·10^{-4}$)	0.94	0.3	$1.5·10^{-6}$	0.11	0.1	0.97	$2.6·10^{-6}$	5.7
**Fluid Intelligence SI**	0.29±0.06 ($3.0·10^{-7}$)	0.03	0.22	$9.2·10^{-5}$	0.12	0.24	0.91	$3.3·10^{-9}$	7.7
**Vocabulary Comprehension**	0.64±0.04 ($2.7·10^{-30}$)	0.68	0.45	$3.5·10^{-5}$	0.24	0.79	0.71	$1.7·10^{-25}$	16.5
**Processing Speed**	0.33±0.06 ($1.0·10^{-7}$)	0.65	0.36	0.24	0.1	0.19	0.53	0.13	0.0
**Verbal Episodic Memory CR**	0.46±0.06 ($2.6·10^{-13}$)	$3.2·10^{-3}$	0.3	0.4	0.55	0.1	0.72	0.21	3.2
**Verbal Episodic Memory RT**	0.33±0.06 ($4.3·10^{-8}$)	0.24	0.76	0.21	0.4	0.97	0.48	$1.1·10^{-5}$	2.6
**Working memory**	0.52±0.05 ($1.1·10^{-18}$)	$6.0·10^{-3}$	0.06	0.53	$6.1·10^{-3}$	0.1	0.29	$4.1·10^{-7}$	4.8
**Episodic Memory**	0.44±0.06 ($3.8·10^{-14}$)	0.21	0.03	$1.3·10^{-4}$	0.93	0.11	0.97	$1.7·10^{-7}$	5.2
**Cognitive Flexibility**	0.38±0.06 ($1.2·10^{-10}$)	0.26	0.43	0.27	0.56	0.3	0.97	$7.9·10^{-3}$	1.0
**Inhibition**	0.31±0.06 ($3.0·10^{-7}$)	0.14	0.32	0.02	0.11	0.49	0.27	0.32	1.7
**Reading Decoding**	0.67±0.04 ($1.2·10^{-30}$)	0.32	0.8	0.01	0.14	0.29	0.63	$1.1·10^{-24}$	15.7

All scores have been measured by HCP. They follow protocols described in the NIH toolbox.

Fluid Intelligence: measured by the Penn Progressive Matrices Test (CR: Number of Correct Responses, SI: Total Skipped Items, RT: Median Reaction Time for CR)

Language/Vocabulary Comprehension: measured by the Picture Vocabulary Test

Processing Speed: measured by the Pattern Comparison Processing Speed Test

Verbal Episodic Memory: measured by the Penn Word Memory Test (CR: Number of Correct Responses, RT: Median Reaction Time for CR)

Working memory: measured by the List Sorting Working Memory Test

Episodic Memory: measured by the Picture Sequence Memory Test

Executive Function/Cognitive Flexibility: measured by the Dimensional Change Card Sort Test

Executive Function/Inhibition: measured by the Flanker Inhibitory Control and Attention Test

Language/Reading Decoding: measured by the Oral Reading Recognition Test

More details can be found at: https://wiki.humanconnectome.org/display/PublicData/HCP+Data+Dictionary+Public-+500+Subject+Release

S2 Table. Genetic (ρ_p (p) / $\rho_g \pm \sigma_g$ (p)) (above diagonal) and phenotypic (below diagonal) correlations for the NIH standard behavioral scores and task variables.

	Language Acc	Language Median RT	Story Acc	Story Median RT	Story Avg Diff level	Math Acc	Math Median RT	Math Avg Diff level	PMAT24 A CR	PMAT24 A SI	PMAT24 A RTCR	PicVocab	ProcSpeed	IWRD TOT	IWRD RTC	ListSort	PicSeq	CardSort	Flanker	ReadEng
**Language Acc**	*	-0.41 ±0.19 (0.03)	0.66 ±0.11 (10^{-3})	-0.06 ±0.19 (0.74)	0.88 ±0.07 (10^{-8})	0.9 ±0.04 (10^{-9})	-0.73 ±0.23 (10^{-4})	0.67 ±0.11 (10^{-5})	0.61 ±0.14 (10^{-5})	-0.53 ±0.16 (10^{-3})	0.22 ±0.17 (0.18)	0.57 ±0.10 (10^{-8})	0.44 ±0.16 (10-3)	0.32 ±0.15 (0.04)	-0.24 ±0.13 (0.07)	0.50 ±0.12 (10^{-5})	0.76 ±0.14 (10^{-9})	0.61 ±0.15 (10^{-5})	0.55 ±0.15 (10^{-4})	0.67 ±0.10 (10^{-10})
**Language Median RT**	-0.15 (10^{-7})	*	-0.18 ±0.27 (0.49)	0.9 ±0.06 (10^{-3})	-0.41 ±0.2 (0.04)	-0.46 ±0.16 (10^{-3})	0.85 ±0.08 (10^{-3})	-0.39 ±0.24 (0.07)	-0.42 ±0.17 (0.01)	0.4 ±0.2 (0.05)	-0.05 ±0.22 (0.8)	-0.2 ±0.15 (0.17)	-0.49 ±0.2 (0.02)	-0.54 ±0.2 (10-3)	0.3 ±0.16 (0.08)	-0.13 ±0.16 (0.41)	-0.27 ±0.17 (0.1)	-0.13 ±0.19 (0.5)	-0.1 ±0.21 (0.62)	0.0 ±0.14 (0.99)
**Story Acc**	0.71 (10^{-160})	-0.01 (0.67)	*	-0.22 ±0.27 (0.39)	0.25 ±0.2 (0.22)	0.27 ±0.18 (0.13)	-0.09 ±0.28 (0.75)	0.98 ±0.12 (10^{-6})	0.33 ±0.18 (0.06)	-0.27 ±0.22 (0.19)	0.18 ±0.23 (0.41)	0.65 ±0.15 (10^{-6})	0.26 ±0.22 (0.22)	0.59 ±0.21 (10-3)	-0.15 ±0.18 (0.38)	0.17 ±0.17 (0.3)	0.52 ±0.21 (10^{-3})	0.45 ±0.2 (0.02)	0.49 ±0.22 (0.02)	0.60 ±0.16 (10^{-5})
**Story Median RT**	-0.06 (0.07)	0.83 (10^{-264})	-0.02 (0.53)	*	0.01 ±0.2 (0.97)	-0.01 ±0.18 (0.95)	0.54 ±0.21 (0.05)	-0.42 ±0.26 (0.06)	-0.27 ±0.17 (0.12)	0.28 ±0.21 (0.17)	0.11 ±0.22 (0.61)	-0.14 ±0.14 (0.33)	-0.37 ±0.2 (0.07)	-0.41 ±0.2 (0.04)	0.2 ±0.17 (0.25)	-0.03 ±0.16 (0.85)	-0.01 ±0.17 (0.94)	-0.12 ±0.19 (0.55)	-0.01 ±0.21 (0.98)	0.17 ±0.13 (0.21)
**Story Avg Diff level**	0.6 (10^{-105})	-0.12 (10^{-5})	0.13 (10^{-5})	-0.07 (0.03)	*	0.98 ±0.05 (10^{-9})	-0.88 ±0.28 (10^{-5})	0.29 ±0.16 (0.09)	0.51 ±0.14 (10^{-4})	-0.55 ±0.17 (10^{-3})	0.14 ±0.18 (0.44)	0.31 ±0.11 (100)	0.31 ±0.16 (0.06)	0.32 ±0.16 (0.05)	-0.24 ±0.14 (0.09)	0.47 ±0.12 (10^{-4})	0.47 ±0.13 (10^{-4})	0.70 ±0.14 (10^{-6})	0.25 ±0.16 (0.14)	0.46 ±0.11 (10^{-5})
**Math Acc**	0.81 (10^{-243})	-0.2 (10^{-11})	0.15 (10^{-7})	-0.06 (0.04)	0.73 (10^{-178})	*	-0.86 ±0.19 (10^{-6})	0.31 ±0.14 (0.03)	0.59 ±0.13 (10^{-6})	-0.57 ±0.15 (10^{-4})	0.21 ±0.16 (0.18)	0.35 ±0.10 (10^{-4})	0.41 ±0.15 (10-3)	0.1 ±0.14 (0.48)	-0.22 ±0.12 (0.07)	0.54 ±0.11 (10^{-6})	0.67 ±0.11 (10^{-8})	0.50 ±0.14 (10^{-4})	0.44 ±0.14 (10^{-3})	0.51 ±0.09 (10^{-7})
**Math Median RT**	-0.19 (10^{-10})	0.81 (10^{-250})	-0.0 (0.95)	0.34 (10^{-31})	-0.13 (10^{-5})	-0.27 (10^{-19})	*	-0.19 ±0.23 (0.4)	-0.49 ±0.19 (10^{-3})	0.43 ±0.22 (0.05)	-0.23 ±0.23 (0.32)	-0.16 ±0.16 (0.29)	-0.46 ±0.23 (0.04)	-0.55 ±0.23 (0.01)	0.34 ±0.17 (0.06)	-0.21 ±0.17 (0.23)	-0.49 ±0.19 (10^{-3})	-0.1 ±0.21 (0.65)	-0.19 ±0.22 (0.39)	-0.22 ±0.15 (0.14)
**Math Avg Diff level**	0.44 (10^{-50})	0.1 (10^{-3})	0.48 (10^{-62})	0.18 (10^{-9})	0.28 (10^{-20})	0.21 (10^{-12})	-0.02 (0.51)	*	0.16 ±0.14 (0.26)	-0.12 ±0.17 (0.49)	-0.19 ±0.18 (0.3)	0.57 ±0.10 (10^{-7})	0.07 ±0.17 (0.67)	0.32 ±0.16 (0.05)	-0.36 ±0.15 (0.01)	0.38 ±0.13 (10^{-3})	0.39 ±0.14 (10^{-3})	0.22 ±0.16 (0.16)	0.45 ±0.17 (10-3)	0.28 ±0.11 (0.01)
**PMAT24 A CR**	0.36 (10^{-33})	-0.12 (10^{-4})	0.16 (10^{-7})	-0.06 (0.05)	0.3 (10^{-24})	0.37 (10^{-35})	-0.13 (10^{-5})	0.17 (10^{-8})	*	-0.97 ±0.02 (10^{-10})	0.62 ±0.09 (10^{-4})	0.51 ±0.08 (10-8)	0.16 ±0.14 (0.26)	0.34 ±0.12 (10-3)	-0.22 ±0.11 (0.05)	0.61 ±0.10 (10^{-9})	0.4 ±0.11 (10^{-4})	0.33 ±0.12 (10^{-3})	0.22 ±0.13 (0.09)	0.44 ±0.08 (10^{-6})
**PMAT24 A SI**	-0.32 (10^{-27})	0.1 (10^{-3})	-0.14 (10^{-5})	0.05 (0.08)	-0.28 (10^{-20})	-0.34 (10^{-30})	0.11 (10^{-4})	-0.15 (10^{-6})	-0.97 (10^{-300})	*	-0.73 ±0.08 (10^{-4})	-0.54 ±0.10 (10^{-7})	-0.21 ±0.17 (0.21)	-0.36 ±0.15 (0.02)	0.3 ±0.13 (0.03)	-0.75 ±0.13 (10^{-9})	-0.36 ±0.13 (10^{-3})	-0.35 ±0.14 (0.02)	-0.23 ±0.16 (0.14)	-0.44 ±0.10 (10^{-5})
**PMAT24 A RTCR**	0.17(10^{-8})	0.01 (0.78)	0.08 (10^{-3})	0.04 (0.17)	0.12 (10^{-4})	0.17 (10^{-8})	-0.03 (0.35)	0.01 (0.73)	0.72 (10^{-193})	-0.7 (10^{-178})	*	0.13 ±0.13 (0.3)	-0.22 ±0.19 (0.24)	0.26 ±0.19 (0.16)	-0.05 ±0.16 (0.77)	0.37 ±0.15 (0.01)	0.06 ±0.15 (0.7)	0.07 ±0.17 (0.68)	-0.07 ±0.18 (0.72)	0.28 ±0.13 (0.03)
**PicVocab**	0.4 (10^{-42})	-0.13 (10^{-5})	0.27 (10^{-19})	-0.09 (10^{-3})	0.32 (10^{-26})	0.33 (10^{-29})	-0.13 (10^{-5})	0.28 (10^{-21})	0.5 (10^{-76})	-0.47 (10^{-67})	0.26 (10^{-19})	*	0.11 ±0.1 (0.31)	0.36 ±0.09 (10^{-4})	-0.24 ±0.09 (10^{-3})	0.39 ±0.08 (10^{-6})	0.08 ±0.09 (0.37)	0.14 ±0.10 (0.13)	0.18 ±0.11 (0.09)	Rhog ERROR
**ProcSpeed**	0.17 (10^{-8})	-0.15 (10^{-7})	0.07 (0.02)	-0.13 (10^{-5})	0.21 (10^{-11})	0.17 (10^{-8})	-0.13 (10^{-5})	0.17 (10^{-8})	0.15 (10^{-7})	-0.14 (10^{-6})	-0.04 (0.18)	0.16 (10^{-8})	*	0.06 ±0.16 (0.72)	-0.46 ±0.12 (10^{-4})	0.22 ±0.12 (0.07)	0.25 ±0.13 (0.06)	0.48 ±0.11 (10^{-3})	0.30 ±0.14 (0.07)	0.01 ±0.11 (0.95)
**IWRD TOT**	0.2 (10^{-11})	-0.08 (0.01)	0.12 (10^{-5})	-0.01 (0.65)	0.18 (10^{-9})	0.18 (10^{-9})	-0.12 (10^{-4})	0.13 (10^{-5})	0.23 (10^{-15})	-0.21 (10^{-13})	0.13 (10^{-6})	0.26 (10^{-20})	0.14 (10^{-7})	*	-0.48 ±0.11 (10^{-4})	0.2 ±0.11 (0.1)	0.47 ±0.12 (10^{-4})	0.3 ±0.14 (0.03)	0.49 ±0.16 (10^{-3})	0.38 ±0.09 (10^{-4})
**IWRD RTC**	-0.1 (10^{-4})	0.21 (10^{-11})	-0.01 (0.77)	0.14 (10^{-6})	-0.16 (10^{-7})	-0.14 (10^{-6})	0.2 (10^{-11})	-0.13 (10^{-5})	-0.09 (10^{-3})	0.08 (10^{-3})	0.08 (10^{-3})	-0.18 (10^{-10})	-0.25 (10^{-19})	-0.28 (10^{-22})	*	-0.35 ±0.10 (10^{-4})	-0.26 ±0.11 (0.02)	-0.15 ±0.12 (0.24)	-0.14 ±0.13 (0.31)	-0.24 ±0.09 (10^{-3})
**ListSort**	0.34 (10^{-30})	-0.12 (10^{-4})	0.16 (10^{-7})	-0.05 (0.09)	0.3 (10^{-23})	0.34 (10^{-30})	-0.14 (10^{-6})	0.2 (10^{-11})	0.36 (10^{-37})	-0.34 (10^{-34})	0.14 (10^{-7})	0.34 (10^{-33})	0.18 (10^{-10})	0.16 (10^{-8})	-0.1 (10^{-4})	*	0.54 ±0.09 (10^{-8})	0.26 ±0.11 (0.02)	0.13 ±0.12 (0.29)	0.38 ±0.08 (10^{-6})
**PicSeq**	0.26 (10^{-18})	-0.11 (10^{-4})	0.14 (10^{-5})	-0.03 (0.38)	0.21 (10^{-12})	0.25 (10^{-17})	-0.16 (10^{-7})	0.17 (10^{-8})	0.3 (10^{-25})	-0.28 (10^{-22})	0.12 (10^{-5})	0.2 (10^{-12})	0.2 (10^{-13})	0.24 (10^{-17})	-0.12 (10^{-5})	0.34 (10^{-35})	*	0.29 ±0.12 (0.02)	0.35 ±0.14 (10^{-2})	0.16 ±0.09 (0.06)
**CardSort**	0.25 (10^{-16})	-0.17 (10^{-8})	0.12 (10^{-4})	-0.12 (10^{-4})	0.26 (10^{-17})	0.25 (10^{-16})	-0.16 (10^{-7})	0.14 (10^{-6})	0.22 (10^{-14})	-0.22 (10^{-15})	0.04 (0.22)	0.19 (10^{-11})	0.42 (10^{-53})	0.15 (10^{-7})	-0.14 (10^{-7})	0.21 (10^{-13})	0.21 (10^{-13})	*	0.50 ±0.11 (10^{-4})	0.30 ±0.09 (10^{-3})
**Flanker**	0.16 (10^{-7})	-0.13 (10-5)	0.07 (0.03)	-0.11 (10^{-4})	0.17 (10^{-8})	0.17 (10^{-8})	-0.11 (10^{-4})	0.13 (10^{-5})	0.13 (10^{-6})	-0.12 (10^{-5})	-0.01 (0.61)	0.2 (10^{-13})	0.39 (10^{-46})	0.08 (10^{-3})	-0.16 (10^{-8})	0.14 (10^{-6})	0.15 (10^{-7})	0.52 (10^{-83})	*	0.14 ±0.11 (0.18)
**ReadEng**	0.46 (10^{-56})	-0.1 (10-3)	0.23 (10^{-13})	-0.01 (0.83)	0.39 (10^{-40})	0.45 (10^{-54})	-0.16 (10^{-7})	0.23 (10^{-14})	0.48 (10^{-70})	-0.45 (10^{-62})	0.3 (10^{-26})	0.7 (10^{-178})	0.16 (10^{-8})	0.28 (10^{-22})	-0.15 (10^{-7})	0.36 (10^{-38})	0.21 (10^{-13})	0.25 (10^{-18})	0.18 (10^{-10})	*

S3 Table. Heritability estimates for MATH activations in the left hemisphere and associated p-values for covariates. Only significant estimates after Bonferroni correction, $p < 1.4 \cdot 10^{-4}$, are presented.

Trait	$h^2 \pm SE(p)$	Age	Age^2	Sex	Age*Sex	Age^2*Sex	Ethni	Educ
		p-val						
**Area 7m**	$0.3 \pm 0.06\ (1.0 \cdot 10^{-6})$	0.55	0.76	0.55	0.58	0.9	0.17	0.49
**Area 8Ad**	$0.26 \pm 0.07\ (3.2 \cdot 10^{-5})$	0.54	0.44	0.89	0.75	0.41	0.52	$9.8 \cdot 10^{-3}$
**Area 8Av**	$0.29 \pm 0.06\ (1.2 \cdot 10^{-6})$	0.94	0.48	0.02	0.86	0.04	0.11	0.15
**Area 8C**	$0.29 \pm 0.06\ (1.6 \cdot 10^{-6})$	0.68	0.25	$3.8 \cdot 10^{-3}$	0.58	0.01	0.65	0.15
**Area FST**	$0.27 \pm 0.06\ (1.0 \cdot 10^{-5})$	0.18	0.37	0.33	0.57	0.1	0.41	0.48
**Area IntraParietal 0**	$0.33 \pm 0.07\ (6.0 \cdot 10^{-7})$	0.62	0.13	0.01	0.73	0.02	0.23	0.59
**Area IntraParietal 1**	$0.29 \pm 0.06\ (2.1 \cdot 10^{-6})$	0.61	0.02	0.04	0.74	$8.8 \cdot 10^{-3}$	$6.1 \cdot 10^{-5}$	0.09
**Area Lateral IntraParietal dorsal**	$0.27 \pm 0.06\ (5.5 \cdot 10^{-6})$	0.83	0.25	0.16	0.72	0.09	$1.2 \cdot 10^{-3}$	0.91
**Area Lateral IntraParietal ventral**	$0.3 \pm 0.06\ (1.4 \cdot 10^{-6})$	0.77	0.64	0.97	0.58	0.51	0.35	0.55
**Area PF opercular**	$0.26 \pm 0.06\ (3.3 \cdot 10^{-5})$	0.12	0.44	0.21	0.83	0.3	0.62	0.33
**Area PGp**	$0.45 \pm 0.06\ (5.2 \cdot 10^{-13})$	0.73	0.78	0.03	0.9	0.52	0.84	0.64
**Area PGs**	$0.3 \pm 0.07\ (2.4 \cdot 10^{-6})$	0.88	0.9	0.55	0.78	0.51	0.17	0.07
**Area TE1 Middle**	$0.28 \pm 0.07\ (1.1 \cdot 10^{-5})$	0.85	0.55	0.41	0.66	0.8	0.05	0.1
**Area TE1 posterior**	$0.25 \pm 0.07\ (1.1 \cdot 10^{-4})$	0.94	0.28	$1.4 \cdot 10^{-3}$	0.92	$4.5 \cdot 10^{-3}$	0.1	0.01
**Area p32 prime**	$0.25 \pm 0.07\ (1.0 \cdot 10^{-4})$	0.59	0.39	0.4	0.79	0.23	0.8	0.69
**Inferior 6-8 Transitional Area**	$0.3 \pm 0.06\ (6.0 \cdot 10^{-7})$	0.72	0.39	0.03	0.99	0.04	0.13	$2.6 \cdot 10^{-4}$
**Lateral Area 7P**	$0.29 \pm 0.06\ (2.9 \cdot 10^{-6})$	0.89	0.36	0.21	0.73	0.1	0.55	0.04
**Medial Area 7A**	$0.29 \pm 0.07\ (1.1 \cdot 10^{-5})$	0.56	0.69	0.85	0.48	0.64	0.93	0.25
**Medial Area 7P**	$0.29 \pm 0.07\ (1.1 \cdot 10^{-5})$	0.51	0.18	0.74	0.23	0.04	0.46	0.89
**Medial IntraParietal Area**	$0.25 \pm 0.07\ (5.6 \cdot 10^{-5})$	0.55	0.49	$7.2 \cdot 10^{-3}$	0.94	0.1	0.16	0.41
**Parieto-Occipital Sulcus Area 1**	$0.26 \pm 0.07\ (7.4 \cdot 10^{-5})$	0.47	0.53	0.27	0.43	0.41	0.94	0.59
**PeriSylvian Language Area**	$0.42 \pm 0.06\ (1.5 \cdot 10^{-10})$	0.3	0.09	0.42	0.83	$5.3 \cdot 10^{-3}$	0.03	0.35
**Superior 6-8 Transitional Area**	$0.23 \pm 0.07\ (1.3 \cdot 10^{-4})$	0.39	0.64	0.13	0.5	0.64	0.49	$5.4 \cdot 10^{-3}$

S4 Table. Heritability estimates for MATH activations in the right hemisphere and associated p-values for covariates. Only significant estimates after Bonferroni correction, $p < 1.4 \cdot 10^{-4}$, are presented.

Trait	h^2±SE(p)	Age	Age^2	Sex	Age*Sex	Age^2*Sex	Ethni	Educ
		p-val						
**Area 46**	0.25±0.07 ($4.9 \cdot 10^{-5}$)	0.51	0.49	0.68	0.9	0.15	0.81	0.79
**Area 47l**	0.25±0.07 ($5.4 \cdot 10^{-5}$)	0.81	0.45	0.96	0.7	0.43	0.76	0.42
**Area 7m**	0.24±0.07 ($1.1 \cdot 10^{-4}$)	0.7	0.43	0.27	0.87	0.29	0.01	0.41
**Area 8C**	0.25±0.07 ($1.3 \cdot 10^{-4}$)	0.18	0.84	0.28	0.35	0.6	0.21	0.9
**Area IntraParietal 0**	0.26±0.07 ($2.5 \cdot 10^{-5}$)	0.87	0.7	0.74	0.08	0.05	0.55	0.76
**Area Lateral IntraParietal dorsal**	0.27±0.07 ($5.5 \cdot 10^{-5}$)	0.82	0.82	0.33	0.49	0.16	0.03	0.44
**Area PGp**	0.29±0.06 ($1.6 \cdot 10^{-6}$)	0.28	0.76	$5.6 \cdot 10^{-3}$	0.81	0.22	0.64	0.32
**Area PGs**	0.25±0.07 ($4.8 \cdot 10^{-5}$)	0.5	0.66	$3.8 \cdot 10^{-3}$	0.55	0.99	0.53	0.75
**Area STGa**	0.26±0.07 ($1.4 \cdot 10^{-4}$)	0.93	0.69	0.98	0.55	0.35	0.17	0.14
**Area STSd posterior**	0.3±0.07 ($3.2 \cdot 10^{-6}$)	0.11	0.55	0.07	0.55	0.91	0.66	0.45
**Area TA2**	0.28±0.07 ($1.2 \cdot 10^{-5}$)	0.4	0.9	0.25	0.25	0.8	0.75	0.59
**Area TE1 Middle**	0.28±0.06 ($6.6 \cdot 10^{-6}$)	0.6	0.44	0.31	0.21	0.83	0.82	0.81
**Area TemporoParietoOccipital Junction 1**	0.29±0.07 ($2.1 \cdot 10^{-5}$)	0.53	0.18	0.07	0.52	0.92	0.27	0.97
**Area TemporoParietoOccipital Junction 2**	0.24±0.07 ($1.0 \cdot 10^{-4}$)	0.18	0.62	0.14	0.52	0.44	0.93	0.36
**Area posterior 9-46v**	0.3±0.06 ($1.7 \cdot 10^{-6}$)	0.68	0.09	$6.7 \cdot 10^{-3}$	0.97	0.02	0.54	0.63
**Area ventral 23 a+b**	0.29±0.07 ($7.4 \cdot 10^{-6}$)	0.92	0.77	$6.7 \cdot 10^{-3}$	0.51	0.63	0.21	0.54
**Auditory 4 Complex**	0.28±0.06 ($3.4 \cdot 10^{-6}$)	0.78	0.63	0.01	0.68	0.8	0.41	0.3
**Auditory 5 Complex**	0.39±0.06 ($1.3 \cdot 10^{-10}$)	0.22	0.11	0.05	0.42	0.07	0.19	0.99
**Dorsal Transitional Visual Area**	0.31±0.07 ($1.7 \cdot 10^{-6}$)	0.33	0.62	0.02	0.96	0.53	0.55	0.79
**Fourth Visual Area**	0.25±0.07 ($6.8 \cdot 10^{-5}$)	0.15	0.07	0.89	0.84	0.02	0.3	0.22
**Inferior 6-8 Transitional Area**	0.24±0.06 ($7.9 \cdot 10^{-5}$)	0.08	0.51	0.32	0.1	0.05	0.15	0.92
**Lateral Area 7P**	0.35±0.07 ($1.0 \cdot 10^{-7}$)	0.59	0.26	0.55	0.56	0.13	0.68	$5.6 \cdot 10^{-3}$
**Lateral Belt Complex**	0.29±0.07 ($2.3 \cdot 10^{-6}$)	0.71	0.1	0.55	0.53	0.12	0.5	0.75
**Medial IntraParietal Area**	0.26±0.07 ($3.5 \cdot 10^{-5}$)	0.53	0.98	0.94	0.06	0.41	0.17	0.25
**ParaBelt Complex**	0.35±0.06 ($2.5 \cdot 10^{-8}$)	0.65	0.43	0.49	0.68	0.16	0.23	0.74
**Parieto-Occipital Sulcus Area 1**	0.32±0.06 ($1.0 \cdot 10^{-7}$)	0.98	0.61	$7.0 \cdot 10^{-3}$	0.31	0.95	0.56	0.43
**PreCuneus Visual Area**	0.29±0.07 ($3.8 \cdot 10^{-5}$)	0.2	0.78	$7.3 \cdot 10^{-3}$	0.78	0.87	0.16	0.35
**Premotor Eye Field**	0.36±0.06 ($2.6 \cdot 10^{-8}$)	0.74	0.64	0.82	0.82	0.46	0.72	0.36
**Primary Auditory Cortex**	0.26±0.07 ($4.5 \cdot 10^{-5}$)	0.99	0.11	0.58	0.6	0.06	$1.1 \cdot 10^{-3}$	0.36

S5 Table. Heritability estimates for STORY activations in the left hemisphere and associated p-values for covariates. Only significant estimates after Bonferroni correction, $p < 1.4\cdot10^{-4}$, are presented.

Trait	h^2±SE(p)	Age	Age^2	Sex	Age*Sex	Age^2*Sex	Ethni	Educ
		p-val						
**Area 45**	0.22±0.06 ($1.4\cdot10^{-4}$)	0.98	0.04	0.59	0.24	0.04	0.49	0.08
**Area 47l**	0.29±0.06 ($1.5\cdot10^{-6}$)	0.76	0.29	0.01	0.17	0.1	0.92	0.37
**Area 7m**	0.26±0.07 ($2.0\cdot10^{-5}$)	0.2	0.64	$4.4\cdot10^{-3}$	0.11	0.64	0.46	0.7
**Area 8Av**	0.28±0.06 ($4.9\cdot10^{-6}$)	0.75	0.14	0.83	0.68	0.02	0.43	0.56
**Area 8C**	0.32±0.06 ($2.1\cdot10^{-8}$)	0.32	0.88	0.03	0.46	0.05	0.96	0.83
**Area PGi**	0.32±0.07 ($9.0\cdot10^{-7}$)	0.21	0.31	0.41	0.86	0.11	0.05	0.11
**Area PGs**	0.24±0.07 ($1.2\cdot10^{-4}$)	0.48	0.35	0.55	0.36	0.12	0.16	0.1
**Area STSd anterior**	0.28±0.06 ($3.0\cdot10^{-6}$)	0.42	0.01	0.13	0.43	$2.7\cdot10^{-3}$	0.51	0.02
**Area STSd posterior**	0.25±0.07 ($7.9\cdot10^{-5}$)	0.33	0.29	0.64	0.59	0.04	0.1	0.12
**Area STSv anterior**	0.28±0.06 ($5.5\cdot10^{-6}$)	0.47	0.03	0.5	0.87	0.02	0.22	0.07
**Area STSv posterior**	0.27±0.06 ($1.0\cdot10^{-5}$)	0.35	0.43	0.25	0.42	0.19	0.08	0.39
**Area TA2**	0.31±0.07 ($1.2\cdot10^{-6}$)	0.17	0.08	$1.8\cdot10^{-3}$	0.97	0.03	0.54	0.49
**Area TE1 Middle**	0.28±0.06 ($6.0\cdot10^{-6}$)	0.33	0.96	0.93	0.62	0.6	0.28	0.04
**Area TE1 anterior**	0.23±0.06 ($1.2\cdot10^{-4}$)	0.02	0.49	0.04	1.0	0.94	0.02	0.93
**Area TE1 posterior**	0.25±0.07 ($5.1\cdot10^{-5}$)	0.92	0.18	0.07	0.37	$6.7\cdot10^{-3}$	0.27	0.07
**Area TemporoParietoOccipital Junction 1**	0.39±0.07 ($1.0\cdot10^{-7}$)	0.16	0.01	0.46	0.63	$1.4\cdot10^{-4}$	0.2	0.03
**Area anterior 47r**	0.24±0.06 ($7.8\cdot10^{-5}$)	0.61	0.4	0.02	0.65	0.05	0.44	0.04
**Area posterior 10p**	0.22±0.06 ($9.5\cdot10^{-5}$)	0.61	0.79	0.57	0.14	0.87	0.43	0.18
**Area posterior 47r**	0.25±0.07 ($9.5\cdot10^{-5}$)	0.12	0.45	0.01	0.22	0.21	0.75	0.5
**Area ventral 23 a+b**	0.28±0.07 ($1.4\cdot10^{-5}$)	0.5	0.95	0.04	0.2	0.45	0.05	0.05
**Inferior 6-8 Transitional Area**	0.23±0.06 ($8.4\cdot10^{-5}$)	0.97	0.2	0.11	0.37	0.05	0.71	$6.0\cdot10^{-3}$
**ParaBelt Complex**	0.24±0.07 ($1.2\cdot10^{-4}$)	0.02	0.1	0.63	0.59	0.05	0.3	0.51
**Parieto-Occipital Sulcus Area 2**	0.26±0.07 ($5.7\cdot10^{-5}$)	0.15	0.56	0.12	0.56	0.29	0.59	0.01
**PeriSylvian Language Area**	0.55±0.05 ($1.3\cdot10^{-17}$)	0.48	0.16	0.89	0.8	0.07	0.06	0.85

S6 Table. Heritability estimates for STORY activations in the *right hemisphere* and associated p-values for covariates. Only significant estimates after Bonferroni correction, $p < 1.4 \cdot 10^{-4}$, are presented.

Trait	h^2±SE(p)	Age	Age^2	Sex	Age*Sex	Age^2*Sex	Ethni	Educ
		p-val						
**Area 45**	0.3±0.07 ($1.8 \cdot 10^{-6}$)	0.76	0.92	0.31	0.9	0.93	0.21	0.18
**Area 47l**	0.31±0.07 ($2.6 \cdot 10^{-6}$)	0.14	0.59	0.22	0.35	0.75	0.84	0.74
**Area 8Av**	0.24±0.07 ($1.0 \cdot 10^{-4}$)	0.46	0.97	0.13	0.69	0.17	0.38	0.15
**Area 8C**	0.27±0.07 ($1.5 \cdot 10^{-5}$)	0.09	0.54	0.24	0.12	0.56	0.52	0.94
**Area 9 Middle**	0.25±0.07 ($7.2 \cdot 10^{-5}$)	0.13	0.75	0.06	0.3	0.75	0.1	0.45
**Area STGa**	0.3±0.07 ($8.1 \cdot 10^{-6}$)	0.93	0.2	0.59	0.99	0.17	0.88	0.06
**Area STSd anterior**	0.28±0.07 ($7.4 \cdot 10^{-6}$)	0.57	0.29	0.02	0.61	0.41	0.91	0.03
**Area STSd posterior**	0.29±0.06 ($1.6 \cdot 10^{-6}$)	0.1	0.77	0.11	0.97	0.81	0.85	0.09
**Area STSv anterior**	0.26±0.06 ($1.7 \cdot 10^{-5}$)	0.2	0.02	0.63	0.87	0.17	0.2	0.16
**Area TA2**	0.36±0.06 ($1.8 \cdot 10^{-8}$)	0.08	0.33	$1.6 \cdot 10^{-3}$	0.36	0.43	0.12	0.09
**Area anterior 9-46v**	0.24±0.06 ($5.2 \cdot 10^{-5}$)	0.14	0.5	0.01	0.61	0.1	0.52	0.05
**Area p32**	0.25±0.07 ($7.0 \cdot 10^{-5}$)	0.93	0.77	0.25	0.46	0.48	0.69	0.24
**Area ventral 23 a+b**	0.34±0.07 ($3.0 \cdot 10^{-7}$)	0.55	0.95	$8.1 \cdot 10^{-4}$	0.27	0.89	0.14	0.43
**Auditory 4 Complex**	0.33±0.06 ($2.0 \cdot 10^{-7}$)	0.62	0.8	0.06	0.48	0.9	0.49	0.87
**Auditory 5 Complex**	0.39±0.06 ($9.1 \cdot 10^{-11}$)	0.22	0.09	0.22	0.64	0.08	0.34	0.76
**Inferior 6-8 Transitional Area**	0.24±0.07 ($1.2 \cdot 10^{-4}$)	0.34	0.54	0.11	0.07	0.21	0.1	0.85
**Lateral Belt Complex**	0.25±0.07 ($3.3 \cdot 10^{-5}$)	0.58	0.05	0.92	0.63	0.08	0.97	0.67
**Medial Belt Complex**	0.27±0.07 ($9.2 \cdot 10^{-5}$)	0.92	0.06	0.2	1.0	0.23	0.25	0.83
**ParaBelt Complex**	0.38±0.06 ($1.8 \cdot 10^{-9}$)	0.38	0.38	0.98	0.46	0.09	0.43	0.34
**Primary Auditory Cortex**	0.27±0.07 ($1.4 \cdot 10^{-5}$)	0.96	0.05	0.45	0.53	0.07	$9.7 \cdot 10^{-3}$	0.56

S7 Table. Bivariate genetic analysis results for areals in the *left hemisphere* with the STORY-MATH contrast median activation and the HCP language task accuracy. ρ_p: phenotypic correlation, ρ_g: shared genetic variance, ρ_e: environment correlation

Trait	ρ_p (p)	ρ_g±SE (p)	ρ_e (p)
**Anterior Ventral Insular Area**	0.13 ($4.8\cdot10^{-5}$)	0.61±0.17 ($3.0\cdot10^{-4)}$	-0.12 (0.11)
**Area 10v**	0.19 ($6.6\cdot10^{-10}$)	0.23±0.15 (0.13)	0.11 (0.12)
**Area 31pd**	0.16 ($3.0\cdot10^{-7}$)	0.47±0.15 ($3.4\cdot10^{-3}$)	0.01 (0.88)
**Area 44**	0.19 ($4.1\cdot10^{-10}$)	0.35±0.13 (0.02)	0.12 (0.08)
**Area 45**	0.25 ($8.0\cdot10^{-16}$)	0.46±0.13 ($9.4\cdot10^{-4}$)	0.11 (0.14)
**Area 47l**	0.2 ($2.1\cdot10^{-11}$)	0.43±0.14 ($2.6\cdot10^{-3}$)	-0.0 (1.0)
**Area 47m**	0.17 ($3.2\cdot10^{-8}$)	0.29±0.14 (0.04)	0.07 (0.28)
**Area 47s**	0.19 ($2.8\cdot10^{-10}$)	0.4±0.13 ($3.6\cdot10^{-3}$)	0.01 (0.93)
**Area 7m**	0.2 ($1.4\cdot10^{-10}$)	0.44±0.14 ($2.2\cdot10^{-3}$)	0.02 (0.75)
**Area 8Av**	0.17 ($2.1\cdot10^{-8}$)	0.39±0.12 ($3.5\cdot10^{-3}$)	0.01 (0.88)
**Area 8BM**	0.2 ($1.2\cdot10^{-10}$)	0.75±0.14 ($8.9\cdot10^{-7}$)	-0.08 (0.27)
**Area 8B Lateral**	0.28 ($4.5\cdot10^{-20}$)	0.73±0.14 ($5.2\cdot10^{-7}$)	0.02 (0.72)
**Area 9 Middle**	0.26 ($1.5\cdot10^{-17}$)	0.44±0.13 ($1.4\cdot10^{-3}$)	0.08 (0.29)
**Area 9 Posterior**	0.13 ($1.5\cdot10^{-5}$)	0.52±0.21 (0.01)	-0.02 (0.76)
**Area 9 anterior**	0.14 ($8.6\cdot10^{-6}$)	0.3±0.14 (0.04)	0.03 (0.68)
**Area IFSp**	0.15 ($1.6\cdot10^{-6}$)	0.42±0.12 ($9.4\cdot10^{-4}$)	-0.04 (0.58)
**Area IntraParietal 0**	-0.13 ($1.9\cdot10^{-5}$)	-0.21±0.12 (0.1)	0.0 (0.97)
**Area PGi**	0.28 ($9.8\cdot10^{-21}$)	0.54±0.13 ($1.4\cdot10^{-4}$)	0.11 (0.14)
**Area PGp**	-0.15 ($9.0\cdot10^{-7}$)	-0.27±0.11 (0.01)	0.06 (0.47)
**Area STGa**	0.18 ($9.2\cdot10^{-9}$)	0.34±0.15 (0.03)	0.08 (0.26)
**Area STSd anterior**	0.19 ($2.1\cdot10^{-10}$)	0.52±0.16 ($1.9\cdot10^{-3}$)	0.06 (0.39)
**Area STSd posterior**	0.24 ($7.8\cdot10^{-15}$)	0.47±0.11 ($1.9\cdot10^{-4}$)	0.09 (0.22)
**Area STSv anterior**	0.22 ($1.0\cdot10^{-12}$)	0.35±0.15 (0.03)	0.12 (0.1)
**Area STSv posterior**	0.23 ($4.3\cdot10^{-14}$)	0.45±0.12 ($6.5\cdot10^{-4}$)	0.05 (0.45)
**Area TE1 anterior**	0.21 ($5.5\cdot10^{-12}$)	0.41±0.15 ($8.5\cdot10^{-3}$)	0.1 (0.15)
**Area TG Ventral**	0.21 ($6.2\cdot10^{-12}$)	0.51±0.14 ($6.6\cdot10^{-4}$)	0.01 (0.92)
**Area TG dorsal**	0.23 ($1.5\cdot10^{-14}$)	0.44±0.15 ($5.1\cdot10^{-3}$)	0.06 (0.37)
**Area anterior 47r**	0.21 ($1.9\cdot10^{-11}$)	0.48±0.14 ($1.2\cdot10^{-3}$)	-0.02 (0.82)
**Area posterior 47r**	0.13 ($1.6\cdot10^{-5}$)	0.44±0.17 (0.01)	-0.03 (0.63)
**Auditory 5 Complex**	0.14 ($7.8\cdot10^{-6}$)	0.54±0.14 ($2.0\cdot10^{-4}$)	-0.05 (0.45)
**Dorsal Transitional Visual Area**	-0.15 ($4.9\cdot10^{-7}$)	-0.16±0.16 (0.33)	-0.05 (0.43)
**Lateral Area 7P**	-0.13 ($4.4\cdot10^{-5}$)	-0.04±0.12 (0.74)	-0.04 (0.54)
**Medial Area 7A**	-0.14 ($5.6\cdot10^{-6}$)	-0.14±0.14 (0.33)	-0.06 (0.43)
**Parieto-Occipital Sulcus Area 2**	-0.26 ($1.5\cdot10^{-17}$)	-0.19±0.12 (0.11)	-0.15 (0.03)
**PeriSylvian Language Area**	0.13 ($1.2\cdot10^{-5}$)	0.47±0.12 ($2.0\cdot10^{-4}$)	-0.13 (0.09)
**Perirhinal Ectorhinal Cortex**	0.14 ($5.2\cdot10^{-6}$)	0.59±0.3 (0.03)	0.0 (0.97)
**RetroSplenial Complex**	-0.15 ($1.6\cdot10^{-6}$)	-0.5±0.16 ($1.9\cdot10^{-3}$)	0.02 (0.71)
**Superior Frontal Language Area**	0.27 ($4.8\cdot10^{-19}$)	0.52±0.11 ($3.4\cdot10^{-5}$)	0.07 (0.37)

S8 Table. Bivariate genetic analysis results for areals in the *right hemisphere* with the STORY-MATH contrast median activation and the HCP language task accuracy. ρ_p: phenotypic correlation, ρ_g: shared genetic variance, ρ_e: environment correlation

Trait	ρ_p (p)	ρ_g±SE (p)	ρ_e (p)
**Area 10v**	0.15 ($1.3\cdot10^{-6}$)	0.32±0.13 (0.02)	0.03 (0.66)
**Area 23c**	-0.17 ($2.0\cdot10^{-8}$)	-0.38±0.15 (0.01)	-0.01 (0.91)
**Area 23d**	-0.12 ($8.8\cdot10^{-5}$)	-0.31±0.17 (0.07)	-0.01 (0.92)
**Area 31a**	-0.16 ($2.2\cdot10^{-7}$)	-0.19±0.14 (0.18)	-0.07 (0.32)
**Area 45**	0.14 ($2.5\cdot10^{-6}$)	0.42±0.15 ($7.1\cdot10^{-3}$)	-0.0 (0.98)
**Area 46**	-0.19 ($3.5\cdot10^{-10}$)	-0.14±0.15 (0.36)	-0.12 (0.08)
**Area 47l**	0.12 ($1.1\cdot10^{-4}$)	0.51±0.14 ($2.3\cdot10^{-4}$)	-0.04 (0.56)
**Area 7m**	0.18 ($1.7\cdot10^{-9}$)	0.48±0.15 ($1.9\cdot10^{-3}$)	0.0 (0.95)
**Area 8B Lateral**	0.16 ($3.3\cdot10^{-7}$)	0.41±0.14 ($4.6\cdot10^{-3}$)	0.0 (0.98)
**Area 9 Middle**	0.14 ($3.9\cdot10^{-6}$)	0.35±0.16 (0.03)	-0.01 (0.9)
**Area IFSa**	-0.13 ($3.6\cdot10^{-5}$)	-0.2±0.14 (0.15)	0.01 (0.9)
**Area IntraParietal 0**	-0.16 ($2.6\cdot10^{-7}$)	-0.33±0.12 ($9.3\cdot10^{-3}$)	0.0 (0.98)
**Area IntraParietal 2**	-0.15 ($2.3\cdot10^{-6}$)	-0.29±0.15 (0.05)	-0.02 (0.8)
**Area PF Complex**	-0.13 ($3.7\cdot10^{-5}$)	-0.22±0.13 (0.1)	-0.03 (0.72)
**Area PFm Complex**	-0.12 ($5.5\cdot10^{-5}$)	-0.09±0.15 (0.55)	-0.08 (0.28)
**Area PGi**	0.12 ($6.5\cdot10^{-5}$)	0.42±0.15 ($8.0\cdot10^{-3}$)	0.02 (0.76)
**Area PGp**	-0.18 ($5.1\cdot10^{-9}$)	-0.4±0.12 ($9.6\cdot10^{-4}$)	0.11 (0.13)
**Area PHT**	-0.14 ($7.3\cdot10^{-6}$)	-0.18±0.14 (0.21)	-0.01 (0.85)
**Area STGa**	0.15 ($2.0\cdot10^{-6}$)	0.24±0.16 (0.14)	0.06 (0.38)
**Area STSd anterior**	0.19 ($4.6\cdot10^{-10}$)	0.4±0.12 ($2.0\cdot10^{-3}$)	0.05 (0.51)
**Area STSd posterior**	0.12 ($1.0\cdot10^{-4}$)	0.27±0.14 (0.05)	0.03 (0.68)
**Area STSv anterior**	0.14 ($2.8\cdot10^{-6}$)	0.36±0.13 (0.01)	-0.0 (0.95)
**Area TE1 anterior**	0.12 ($6.8\cdot10^{-5}$)	0.32±0.15 (0.04)	0.07 (0.32)
**Area TG dorsal**	0.19 ($9.8\cdot10^{-10}$)	0.65±0.17 ($1.5\cdot10^{-4}$)	-0.02 (0.81)
**Area anterior 10p**	-0.14 ($5.2\cdot10^{-6}$)	-0.21±0.18 (0.24)	-0.05 (0.45)
**Area anterior 9-46v**	-0.12 ($7.7\cdot10^{-5}$)	0.05±0.15 (0.73)	-0.1 (0.13)
**Area p32**	-0.15 ($1.2\cdot10^{-6}$)	-0.49±0.2 ($9.7\cdot10^{-3}$)	0.04 (0.5)
**Dorsal Transitional Visual Area**	-0.15 ($7.4\cdot10^{-7}$)	-0.2±0.15 (0.2)	-0.02 (0.73)
**Frontal OPercular Area 4**	-0.12 ($4.9\cdot10^{-5}$)	0.02±0.19 (0.93)	-0.09 (0.18)
**Lateral Area 7P**	-0.15 ($9.8\cdot10^{-7}$)	-0.32±0.12 ($9.9\cdot10^{-3}$)	0.08 (0.29)
**Medial Area 7A**	-0.16 ($1.2\cdot10^{-7}$)	-0.34±0.17 (0.05)	0.02 (0.76)
**Medial Area 7P**	-0.19 ($6.0\cdot10^{-10}$)	-0.21±0.15 (0.18)	-0.05 (0.45)
**Middle Insular Area**	-0.12 ($5.9\cdot10^{-5}$)	-0.0±0.19 (0.98)	-0.1 (0.12)
**Parieto-Occipital Sulcus Area 1**	-0.12 ($1.0\cdot10^{-4}$)	-0.17±0.12 (0.15)	0.04 (0.59)
**Parieto-Occipital Sulcus Area 2**	-0.26 ($7.7\cdot10^{-18}$)	-0.34±0.12 ($5.5\cdot10^{-3}$)	-0.06 (0.39)
**PeriSylvian Language Area**	-0.15 ($6.4\cdot10^{-7}$)	-0.34±0.18 (0.05)	-0.03 (0.67)
**PreCuneus Visual Area**	-0.14 ($2.8\cdot10^{-6}$)	-0.54±0.18 ($1.5\cdot10^{-3}$)	0.1 (0.13)
**RetroSplenial Complex**	-0.13 ($2.0\cdot10^{-5}$)	-0.28±0.16 (0.07)	0.01 (0.88)
**Superior Frontal Language Area**	0.13 ($3.6\cdot10^{-5}$)	0.49±0.15 ($1.1\cdot10^{-3}$)	-0.05 (0.5)
